# Safety and efficacy of low intensity shockwave (LISW) treatment in patients with erectile dysfunction

**DOI:** 10.1590/S1677-5538.IBJU.2014.0386

**Published:** 2015

**Authors:** A. Ruffo, M. Capece, D. Prezioso, G. Romeo, E. Illiano, L. Romis, G. Di Lauro, F. Iacono

**Affiliations:** 1 Department of Urology, Federico II University, Naples, Italy; 2 Department of Urology, Hospital Santa Maria delle Grazie, Naples, Italy

**Keywords:** Erectile Dysfunction, Therapeutics, Lithotripsy

## Abstract

The primary goal in the management strategy of a patient with ED would be to determine its etiology and cure it when possible, and not just to treat the symptoms alone. One of the new therapeutic strategies is the use of low intensity extracorporeal shockwave (LISW) therapy. The mechanism of shockwave therapy is not completely clear. It is suggested that LISW induces neovascularization and improvement of cavernosal arterial flow which can lead to an improvement of erectile function by releasing NO, VEGF and PCNA.

**Materials and Methods:**

31 patients between February and June 2013 with mild to severe ED and non-Phosphodiesterase 5 inhibitors responders were enrolled. Patients underwent four weekly treatment sessions. During each session 3600 shocks at 0.09mJ/ mm^2^ were given, 900 shocks at each anatomical area (right and left corpus cavernosum, right and left crus). Improvement of the erectile function was evaluated using the International Index of Erectile Function (IIEF-EF), the Sexual Encounter Profile (SEP) diaries (SEP-Questions 2 and 3) and Global Assessment Questions (GAQ-Q1 and GAQ-Q2).

**Results:**

At 3-month follow-up IIEF-EF scores improved from 16.54±6.35 at baseline to 21.03±6.38. Patients answering ‘yes’ to the SEP-Q2 elevated from 61% to 89% and from 32% to 62% in the SEP-Q3. A statistically significant improvement was reported to the Global Assessment Questions (GAQ-Q1 and GAQ-Q2).

**Conclusion:**

In conclusion, we can affirm that LISW is a confirmed therapeutic approach to erectile dysfunction that definitely needs more long-term trials to be clarified and further verified.

## INTRODUCTION

Erectile dysfunction (ED) is the main complaint in male sexual medicine and is defined as the persistent inability to attain and maintain an erection sufficient to permit satisfactory sexual performance. Although ED is a benign disorder, it may affect physical and psychosocial health and may have a significant impact on the quality of life (QoL) of patients and their partners (1).

ED seems to affect 52% of 40-70-year-old men (2). Advances in basic and clinical research on ED during the past 15 years have led to the development of a variety of new treatment options, including pharmacological agents for intracavernous, intraurethral, and oral use and the use of vacuum erection devices (1).

Oral therapies have changed the diagnostic and therapeutic approach to ED becoming a major tool in treating ED. In fact, phosphodiesterase-5 inhibitors (PDE5-i) in the late 1990s and early 2000s completely revolutionized the field of sexual medicine becoming the most popular treatment and the first-line monotherapy for ED (3).

Unfortunately, they are limited for being used before the sexual act and do not modify the physiologic mechanism of penile erection (4).

After the initial enthusiasm of the use of the PDEi, the psychological impact–artificiality of erections and planning for sexual intercourse as well as a not proven curative effect (5) have slightly limited the use of these drugs, leaving the field open to the development of new therapies to treat or maybe cure patients with ED. Furthermore, the frequently reported side-effects of PDE5i, such as headache, dyspepsia, muscular pains, and hot flushes can affect a normal sexual intercourse (6).

The primary goal in the management strategy of a patient with ED would be to determine its etiology and cure when possible, and not just the treatment of symptoms. One of the new therapeutic strategies is the use of low intensity extracorporeal shockwave (LISW) therapy.

Shockwaves (SWs) are longitudinal acoustic waves that travel in the speed of water in ultrasound through body tissue and that carry energy (7). SWs have been widely used in urology to treat urinary stone disease (8), and less often in Peyronie's disease (9) or chronic pelvic pain syndrome (CPPS) in males (10).

The mechanism of action of low-intensity shock waves (LISW) is still not very clear. Many authors suggested that LISW improves erectile function increasing cavernous blood flow and inducing a neovascularization (11). Neovascularization is promoted by the expression of angiogenesis-related growth factors, such as endothelial nitric oxide synthase (NOS), vascular endothelial growth factor (VEGF), and endothelial cell proliferation factors, e.g., proliferating cell nuclear antigen (PCNA) (12).

The aim of our study is to evaluate the improvement of erectile function after therapy with LISW in men affected by mild to moderate ED.

## MATERIALS AND METHODS

### 

#### Study population

31 patients between February and June 2013 with mild to severe ED, and non-Phosphodiesterase 5 inhibitors responders were assessed for this study. Only 2 (6.4%) underwent treatment with PDE5-i in the last four weeks before starting the treatment (Table-1). They all signed an informed consent.

**Table 1:** The pretreatment characteristics of population.

Variable		Patients	P value
**Age (years)**			0.39
	Mean±SD	59.93±12.16	
	N.of subjects analysed	31	
**Time suffering from ED (yrs)**			0.50
	Mean±SD	3.66±4.57	
	N.of subjects analysed	31	
**Treatment with PDE5-I in the last 4 weeks (%)**		6.45	0.12
Proportion		2/31	

Inclusion criteria were: good general health, ED for at least six months, IIEF-EF between 7 to 24 (=mild to moderate).

Exclusion criteria included: neurological pathology, past radical prostatectomy or extensive pelvic surgery, recovering from cancer during the last year, any unstable medical, psychiatric disorder, spinal cord injury, penile anatomical abnormalities, clinically significant chronic hematological disease, anti-androgens or radiotherapy treatment of the pelvic region.

The medical and psychosexual history of all patients were evaluated at baseline to detect comorbidities. Table-2 summarizes the patients’ organic co-morbidities: cardiovascular diseases in 7 pts (22%), hypertension in 18 pts (58%), diabetes in 12 pts (38%) and abnormal total serum cholesterol in 13 pts (41%).

**Table 2:** Analysis of self-reported measures at baseline, 1-month and 3-month follow up by treatment cohort.

Variable	Baseline	Follow-up 1 month	p value	Follow-up 3 months	P value
**IIEf – EF**	16.54±6.35	21.13±6.31	P=0.0075	21.03±6.38	p=0.0096
**SEP-Q_2_ (%)**	61 (yes)	86 (yes)	P=0.0292	89 (yes)	P=0.0112
	38 (no)	13 (no)		10 (no)	
		2 drop-out			
**SEP-Q_3_ (%)**	32 (yes)	58 (yes)	P=0.0402	62 (yes)	P=0.0207
	67 (no)	41 (no)		37 (no)	
		2 drop-out			

(**IIEF-EF**): International Index of Erectile Function; (**SEP-Q2**): Sexual Encounter Profile-Q2; (**SEP-Q3**): Sexual Encounter Profile-Q3

#### Study design

This is a pilot clinical study evaluating safety and efficacy of LISW treatment (performed with Renova ®) on symptomatic ED patients versus baseline.

#### Study schedule

##### a) screening

Patients were visited (visit 1) and those who were using PDE5-i had to go to a flush-out period of three weeks before starting the treatment. Furthermore, they committed to refrain from usage of PDE5-i during the duration of the treatment session.

##### b) Treatment

Patients underwent four weekly treatment sessions. During each session 3600 shocks at 0.09 mJ/mm2 were given. Shocks were applied at the penis shaft at right corpus cavernosum and left corpus cavernosum, right crus and left crus, 900 shocks at each area.

The treatment areas were the same for every session, so that at the end of the full treatment (four sessions) each area received 3600 shocks at an average 0.09mj/mm. We used this protocol under the guidance of Direx Group LTD.

LISW utilize low energy-0.09mJ/mm^2^-equivalent to 10% of the energy used by conventional kidney stone lithotripters in the treatment of urinary tract stones. This device generates a low intensity shockwave focused along a line of 70mm and hence is able to apply shockwaves to the corpora cavernosa and crura effectively.

For the past 3 years, a similar LISW technique has been used in different sites using the same level of energy density to treat ED (13). Shockwaves are created by a special generator and are focused using a specially designed shockwave applicator apparatus. The shockwaves are delivered through the applicator covering the entire corpora cavernosa of the penis.

The treatment does not inflict pain and does not require any anesthesia or sedation.

Each session lasts approximately 30 minutes.

##### c) Primary efficacy objective

To evaluate the increase of number of points in the International Index of Erectile Function (IIEF-EF) questionnaire from baseline (visit 1) to 1 and 3 months after treatment regarding the severity of the symptoms according to minimal clinically important differences in the erectile function domain of the IIEF scale (14). The IIEF-EF was chosen as primary clinical efficacy assessment tool in this study. It has been reported to be brief and reliable, psychometrically sound, and easy to administer in both research and clinical settings. It is available (and cross-culturally validated) in 10 languages and demonstrates adequate sensitivity and specificity for detecting treatment-related changes in erectile function (15).

##### d) Secondary efficacy objective

To study the clinical efficacy of LISW in terms of improvement in sexual activity leading to optimal penetration at 1 and 3 months post-treatment by using the Sexual Encounter Profile (SEP) diaries (SEP-Questions 2 and 3). Patients recorded efficacy information after each sexual encounter by answering the two yes/no questions of the test: SEP Question 2:“Were you able to insert your penis into your partner's vagina?” and SEP Question 3:“Did your erection last long enough for you to have successful intercourse?”.

In addition, patients underwent further evaluation with the Global Assessment Question (GAQ) by answering the two yes/no questions of the test: (GAQ-Q1) “Over the past four weeks has the treatment you have been taking improved your erectile function?” and (GAQ-Q2) “If yes, has the treatment improved your ability to engage in sexual activity over the past four weeks”.

### Statistical analysis

Statistical analysis was performed by the program Statistical Package for Social Sciences for Windows, version 11.5.1 (SPSS Inc., Chicago, IL, USA), using X^2^ test and T-student for categorical data comparisons.

## RESULTS

**Figure 1:**
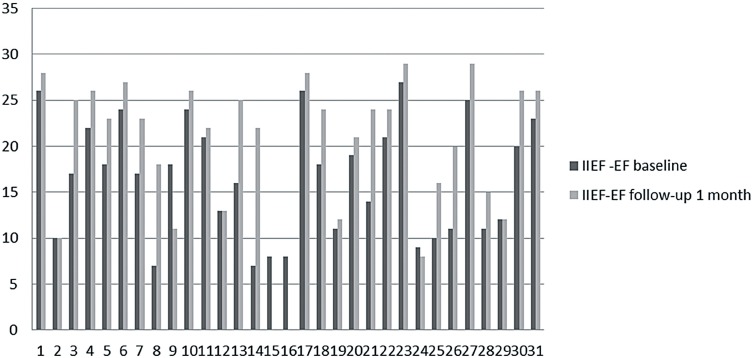
IIEF-EF score at baseline and after 1 month follow-up

**Figure 2:**
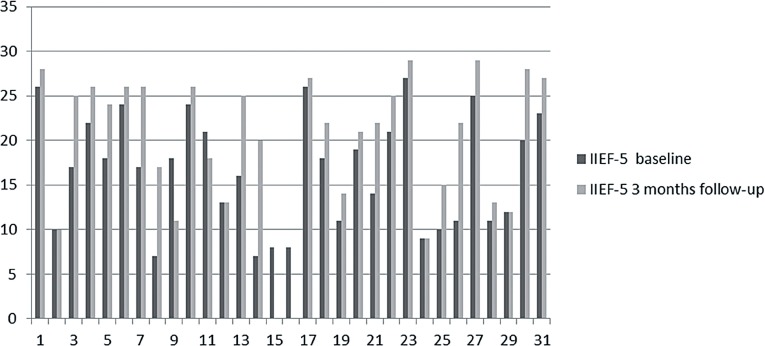
IIEF-5 score at baseline and after 3 month follow-up

**Figure 3:**
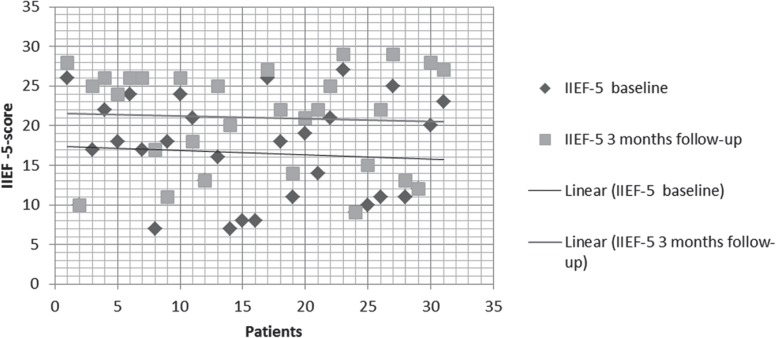
Dispersion date IIEF score baseline and 3 month follow-up

All patients had mild to severe ED at least six months, were non PDE-5i responders, with a mean age of 59.93±12.16 years. Median follow-up was of 3 months (range 2-5 months). Global patient perceptions after treatment with LISW significantly improved. Indeed IIEF-EF score showed significant improvement (baseline 16.54±6.35 vs 21.13±6.31 after 1 month P=0.0075; baseline 16.54±6.35 vs 21.03±6.38 after 3 months p=0.0096) (Table-2; Figure 1–3). About 86% (P=0.0292) and 89% (P=0.0112) of patients answered with a positive answer to SEP Q2 question (“Were you able to insert your penis into your partner's vagina?”) 1 month and 3 months after treatment, respectively, versus 61% positive answers pre-treatment (Table-2). SEP Q3 question (“Did your erection last long enough for you to have successful intercourse?”) was answered positively by 58% (P=0.0402) 1 month after LISW treatment and 62% (P=0.0207) after 3 months. After 1 month of treatment there were two drop-outs (Table-2). Table-3 shows patients’ satisfactions of treatment with GAQ-Q1 (“Over the past four weeks has the treatment you have been taking improved your erectile function?”) and GAQ-Q2 questions (“If yes, has the treatment improved your ability to engage in sexual activity over the past four weeks”). Regarding the individual answers for the GAQ questions, we noticed that 89% and 62% of patients at 1 and 3 months respectively answered “Yes” to the GAQ-Q1 while in the same period 79% and 76% of patients answered “Yes” to the GAQ-Q2 demonstrating success with the treatment (Table-3).

**Table 3:** Analysis of self-reported measures at 1-month and 3-month follow up by treatment cohort.

Variable	Follow-up 1 Month	Follow-up 3 Month	P value
GAQ-Q_1_ (%)	89 (yes)	62 (yes)	P=0.141
	10 (no)	38 (no)	
	2 droup-out	2 droup-out	
GAQ-Q_2_ (%)	79 (yes)	76 (yes)	P=0.7259
	20 (no)	24 (no)	
	2 droup-out	2 droup-out	

**(GAQ-Q1)**: Global Assessment Question- Q1; **(GAQ-Q2)**: Global Assessment Question- Q2

No adverse events were reported during and following treatment.

## DISCUSSION

According to others author's data LISW appears to be significantly effective for increasing erectile function thanks to the improvement in penile hemodynamics (13, 11). By releasing neo-angiogenic factors and subsequent neovascularization of the treated tissue, LISW therapy leads to tissue regeneration (16). In fact, it has been shown that this low intensity energy acts on vascularization inducing a non-enzymatic production of physiologic amounts of nitric oxide (17). Nitric oxide (NO), the smallest known signaling molecule, is produced by three isoforms of NO synthase (NOS; EC 1.14.13.39). Neuronal NOS (nNOS, NOS I) is constitutively expressed in central and peripheral neurons and in some other cell types. Its functions include synaptic plasticity in the central nervous system (CNS), central regulation of blood pressure, smooth muscle relaxation, and vasodilatation via peripheral nitrergic nerves. Nitrergic nerves are of particular importance in the relaxation of corpus cavernosum and penile erection (18). In corpus cavernosum nNos-derived NO activates guanylyl cyclase which synthesizes cyclic GMP (cGMP) from GTP which in turn is the basis for the pro-erectile function of PDE5 inhibitors (19).

The most important isoform is eNOS, which keeps blood vessels dilated, controls blood pressure, and has numerous other vasoprotective and anti-atherosclerotic effects inhibiting DNA synthesis, mitogenesis, and proliferation of vascular smooth muscle cells as well as smooth muscle cell migration. eNOS is mostly expressed in endothelial cells and synthesizes NO in a pulsatile manner (20).

eNOS appears to be a homeostatic regulator of numerous essential cardiovascular functions: in fact, eNOS-derived NO causes vasodilation in all types of blood vessels by stimulating soluble guanylyl cyclase and increasing cyclic GMP in smooth muscle cells that regulates the activity of calcium channels as well as intracellular contractile proteins that affect the relaxation of corpus cavernosum smooth muscle (21). Qiu et al. reported that LISW can partially ameliorate Diabetes Mellitus (DM)-associated ED in rat model by promoting regeneration of nNOS-positive nerves, endothelium, and smooth muscle in the penis. These beneficial effects appear to be mediated by recruitment of endogenous mesenchymal stem cells (MSCs) (22). Wang and colleagues discovered that LISW stimulates the expression of angiogenesis-related growth factors, such as endothelial nitric oxide synthase (eNOS) and vascular endothelial growth factor (VEGF), and endothelial cell proliferation factors, such as proliferating cell nuclear antigen (PCNA).

The eNOS and VEGF began to rise in as early as one week and remained high for 8 weeks, then declined to baseline in 12 weeks; whereas the increase of PCNA and neo-vessels began in 1 week and persisted for 12 weeks and longer (12).

The effect of LISW on intracellular VEGF levels in human umbilical vein endothelial cells (HUVECs) has also been reported by Nishida et al. (23), who found that LISW significantly increased the expression of VEGF mRNA and its receptor, Flt-1. Their studies on the effects of LISW on a porcine model of chronic myocardial ischemia also showed that VEGF expression was significantly upregulated in the ischemic myocardial cells after treatment inducing neovascularization and improving myocardial perfusion (24).

Furthermore, it has been proved that SW therapy improved symptoms and myocardial perfusion in patients with severe coronary artery disease without any complications or adverse effects (24–26).

Regarding erectile dysfunction, Vardi et al. have been the first ones to believe in the use of LISW to improve male sexual function (27). In the first randomized, double-blind, sham-controlled study, they demonstrated a positive short-term clinical and physiological effect on the erectile function of men who respond to oral PDE5Is (28). In another trial they reported an improvement in penile hemodynamics and endothelial function, as well as IIEF-EF domain score in severe ED patients who were poor responders to PDE5Is.

In this paper we demonstrated the efficacy of LISW in the medical management of ED. Our data show a statistically significant improvement of IIEF-EF score (5 points) and an increase of SEP and GAQ scores after treatment.

Limitations of this study are the lack of a sham controlled arm and the relatively low number of participants.

## CONCLUSIONS

LISW has a well-documented positive clinical and physiological effect on erectile function. The preliminary data at 1 and 3 months follow-up are very encouraging and indicate a therapeutic success of this second generation technology for treating ED with linear low-intensity shockwaves. We also noticed that this treatment is feasible and easy to administer and with no side effects reported. Clearly, we cannot assure the long-term efficacy of LISW, so further studies are needed in order to strengthen these results and to assess whether is possible to repeat cyclically the treatment.
